# Concealed Pulmonary Vein Bigeminy during Sinus Rhythm in Patients with Paroxysmal Atrial Fibrillation: A Useful Marker for Pulmonary Vein Firing

**DOI:** 10.1155/2018/1834514

**Published:** 2018-12-10

**Authors:** Jiqiang Hu, Wu Kuang, Xiaoyun Cui, Yan Li, Yang Wu, Qian Lin, Xuan Wang

**Affiliations:** Department of Cardiology, Dongfang Hospital, Beijing University of Chinese Medicine, Fanggu Road, Beijing 100078, China

## Abstract

**Introduction:**

A concealed pulmonary vein (PV) bigeminy (cPVB) may be found in some patients with atrial fibrillation (AF) during sinus rhythm (SR). The aim of this study was to investigate whether the presence of cPVB during SR is associated with a higher PV firing.

**Methods and Results:**

Seven hundred seventy-six PVs (excluding 5 right middle PVs and 8 left common trunks) were mapped in 198 patients with paroxysmal AF (PAF) who underwent circumferential PV isolation. cPVB with a mean coupling interval of 136 ± 16 ms during SR was observed prior to ablation in 22 (11%) patients. Focal firing was provoked prior to ablation in 144 (19%) PVs. The incidence of focal firing was greater in PVs exhibiting cPVB compared with PVs without cPVB (89% vs. 16%; *P* < 0.001). Also, the number of radiofrequency applications required for isolation was greater in ipsilateral PVs, exhibiting cPVB compared with ipsilateral PVs without cPVB (21.6 ± 6.8 vs. 18.2 ± 5.6; *P*=0.024). During a follow-up of 32 ± 20 months, the single ablation success rate was 82%. Compared with patients without cPVB, patients with cPVB were associated with higher recurrence rate of AF (27% vs. 17%; *p*=0.032).

**Conclusion:**

cPVB during SR was observed prior to index ablation in 11% of PAF patients. Such a potential itself may be a PV firing in a concealed manner, which does not reactivate LA. The PV exhibiting cPVB required a greater number of radiofrequency applications for isolation. Compared to patients without cPVB, the recurrence rate of AF in patients with cPVB was greater.

## 1. Introduction

The triggers initiating atrial fibrillation (AF) originate most often from sleeves of left atrial myocardium extending onto the pulmonary veins (PVs) or the PV antrum [1–6]. It is for this reason that electrical isolation of the PVs forms the cornerstone for catheter ablation of AF. A single PV potential closely following or fused with an atrial potential in the ostium of the PVs is the most commonly observed pattern during sinus rhythm (SR) [7, 8]. Isolated reports also showed that a concealed PV bigeminy (cPVB) or an unusual double PV potential (PVP) at the PV ostium could be found in some patients with AF during SR, and focal ablation or isolation of related PV can cure AF [9–11]. However, whether these observations are applicable to a larger series is unknown. Such cPVB may reflect triggered activity in PV or reentry in PV which plays an important role in the PV firing, or may simply be a phenomenon without clinical implication. Therefore, the aim of the study is to investigate whether the presence of cPVB during SR is associated with a higher PV firing and clinical outcomes after the AF ablation.

## 2. Methods

One hundred ninety-eight consecutive drug refractory paroxysmal AF (PAF) patients (mean age 66 ± 20 years, 62% males) who underwent circumferential PV isolation were included in this study from July 2014 to June 2017. To minimize the influence of known clinical predictors, only patients with PAF were included.

### 2.1. Electrophysiologic Study and Catheter Ablation

After obtaining written informed consent, all patients underwent an electrophysiologic study in the fasting state. The antiarrhythmic agents were stopped for at least five half-lives prior to the ablation procedure. Amiodarone was discontinued for at least three months. Before the procedure, cardiac spiral computerized tomography scans were performed to visually define the anatomy of the left atrium (LA) and PVs. All patients underwent transthoracic and transesophageal echocardiography to evaluate the left atrial thrombus and size.

Our technique used in this study has been described in our previous study [12]. Coronary sinus was mapped with a decapolar catheter inserted via the right internal jugular vein. Double separate transseptal punctures were performed. After transseptal puncture, anticoagulation with unfractionated heparin was begun to maintain an activated clotting time above 350 seconds, and selective multiview pulmonary venograms were obtained. To validate mapping, a circular decapolar catheter (Lasso, Biosense Webster) placed around the PV ostium was used. A 3D electroanatomic reconstruction of the LA was made guided by a 3D navigation system (CARTO, Biosense Webster) with a mapping/ablation catheter (Navistar ThermoCool, Biosense Webster) or a Lasso catheter. Before ablation, cPVB was recorded by using the circular mapping catheter in each vein during SR. If the initial rhythm was AF, the patient was cardioverted and then for following 5 minutes. After assessment of cPVB, the circular mapping catheter and mapping/ablation catheter were positioned sequentially into all PVs to identify the PVs exhibiting firing (at least three rapid consecutive ectopies with or without activating the LA). The methods used to provoke PV firing included the use of isoproterenol (3–10 *µ*g/min) combined with cardioversion of sustained AF. During the whole procedure, the heart rate was recorded for further analysis.

Catheter ablation was performed to encircle the right- and left-sided PV in pairs until ipsilateral PVs' isolation was achieved. Septal and lateral continuous circular lesions around the ipsilateral PVs were deployed about 1 cm posterior and 5 mm anterior from their ostia as defined by PV angiography and the 3D map. The circular mapping catheter or ablation catheter was then advanced deeper into the PVs to exclude residual PVPs. After ablation, isoproterenol (3–10 *µ*g/min) was repeated to provoke firing within the atria and isolated PVs. A waiting time >30 minutes was respected.

### 2.2. Definition of cPVB

**Figure 1:**
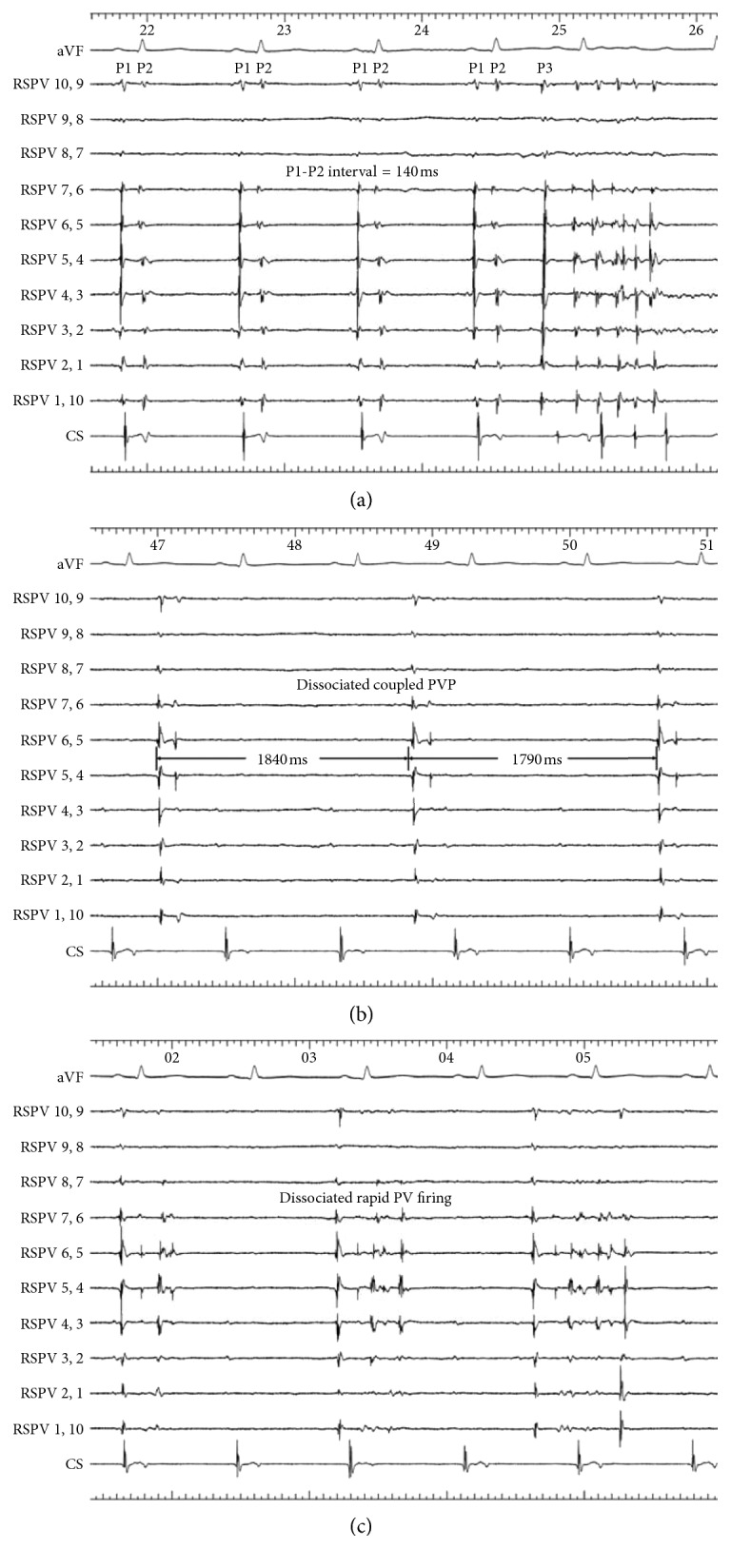
Tracings are from ECG lead aVF and intracardiac electrograms recorded from the electrode pairs of the circular mapping catheter positioned at the ostium of the right superior pulmonary vein (RSPV) and coronary sinus (CS). (a) There is a small atrial potential, followed closely by a pulmonary vein potential (PVP) (*P*1) and then by a second PVP (*P*2) during sinus rhythm. The concealed pulmonary vein bigeminy interval is 140 ms. Spontaneous rapid PV firing with the earliest potential *P*3 conducts out of the vein and activates the atrium. (b) After ipsilateral right pulmonary veins isolation, dissociated coupled pulmonary vein potential was confined in the RSPV. (c) After ipsilateral right pulmonary veins isolation, rapid PV firing was confined in the RSPV with dissociation of atrial potentials.

A Lasso catheter placed at the PV ostium was used to record the presence and characteristics of cPVB. cPVB was defined as wide double PV potentials with an interval >120 ms following far-field LA and right atrial potentials at the PV ostium during SR for at least 15 min in a continuous form before ablation (Figure 1).

### 2.3. Follow-Up

All patients were followed-up at 1 and 3 months and thereafter every 3 months after discharge. Standard 12-lead electrocardiogram and 24-hour Holter recordings were examined routinely. All patients were instructed to contact us with any symptoms or documented AF recurrences. Recurrent AF was defined as any occurrence of sustained atrial tachyarrhythmia lasting at least 30 seconds after a postablation 3-month blanking period.

### 2.4. Redo Ablation Procedure

All patients with AF recurrences were offered redo ablation procedures after the postablation blanking period. Redo ablation procedure was started as described above. After baseline mapping of the PVs, a new CARTO-guided 3D electroanatomical map of the LA was constructed.

### 2.5. Statistical Analysis

Continuous data are presented as mean ± standard deviation. Categorical variables are expressed as number (%). Groups were compared using an unpaired *t*-test, Fisher's exact test, or the chi-square test, as appropriate. Statistical significance was established at a *P* value of <0.05.

## 3. Results

Mapping and ablation were performed in 789 PVs (198 patients): 198 right superior PVs (RSPVs), 198 right inferior PVs (RIPVs), 5 right middle PVs (RMPVs), 190 left superior PVs (LSPVs), 190 left inferior PVs (LIPVs), and 8 left common trunks. Complete isolation (elimination of all PVPs) was obtained in all 789 PVs. For comparison between the 4 principal PVs, the data from the 5 RMPVs and 8 left common trunks were eliminated, leaving 776 PVs for analysis. With the exception of one right femoral pseudoaneurysm and one right internal jugular hematoma, no major complication was found.

### 3.1. Incidence of cPVB during Sinus Rhythm

cPVB during SR was seen prior to ablation in 22 (11%) patients. cPVB was seen in 27 of 776 (3.5%) PVs targeted at the index procedure with a mean of 1.2 cPVB per patient. The characteristics of the cPVB are shown in Table 1. The mean interval between the two PV potentials, the wide double potential interval, was 136 ± 16 ms. Atrial pacing at cycle lengths <400 ms suppressed the second PV potentials in all patients. The cPVB was more common in the superior pulmonary veins compared with inferior veins (LSPV 37%, RSPV 33%, LIPV 19%, and RIPV 11%). The heart rate during sinus rhythm was higher in the patients with cPVB compared with the patients without cPVB (72 ± 8 beats/min vs. 68 ± 8 beats/min; *P*=0.0019) (Table 2).

### 3.2. Relation between PV Firing and cPVB

Focal firing was provoked prior to ablation in 144 (19%) PVs. Twenty-four (89%) PVs exhibiting cPVB during SR showed focal firing spontaneously or with use of isoproterenol combined with cardioversion of sustained AF. The rate of focal firing was greater in PVs exhibiting cPVB compared with PVs without cPVB during SR (89% vs. 16%; *P* < 0.001). Besides, the number of radiofrequency applications required for isolation was greater in ipsilateral PVs exhibiting cPVB compared with ipsilateral PVs without cPVB (21.6 ± 6.8 vs. 18.2 ± 5.6; *P*=0.024) (Table 2).

The second PVP was coupled to the first PVP during SR and disappeared immediately after isolation of PV. Dissociated PVP originating from PV musculature was found in 290 (37%) PVs. All PVs exhibiting cPVB during SR prior to ablation showed dissociated PVP after isolation with a mean interval of 2.4 ± 0.6 second, and 50% of these PVs also showed coupled PVP and rapid PV firing (Figure 1). However, the incidence of dissociated PVP after isolation in PV not exhibiting cPVB prior to ablation was lower than that of PV exhibiting cPVB (35% vs. 100%; *P* < 0.001).

### 3.3. Follow-Up and Redo Ablation Procedure Findings

During a follow-up of 32 ± 20 months, 162 (82%) of the 198 patients were free of sustained atrial tachyarrhythmia lasting more than 30s. Eighty-two percent of the patients with cPVB were off antiarrhythmic drugs compared with 85% in the patients without cPVB, *P*=0.356. All 36 patients with symptomatic recurrence presented with AF or atrial flutter. Compared to that of patients without cPVB, the recurrence rate of AF or atrial flutter in patients with cPVB was greater (27% vs. 17%; *P*=0.032). Also, the basic clinical characteristics showed no difference between these two groups (Table 2).

Twenty of 36 patients underwent a redo procedure, and LA-PV reconnection was found in all patients with a mean of 2.4 ± 0.8 PV per patient (LSPV 25%, LIPV 25%, RSPV 23%, and RIPV 27%). Three patients with cPVB prior to the index procedure underwent a redo procedure. In all these 3 patients, 7 of 8 (88%) PVs with cPVB demonstrated LA-PV reconnection and at least one PV with cPVB showed LA-PV reconnection in each patient.

## 4. Discussion

To our knowledge, this is the first study to demonstrate that the cPVB is associated with PV firing. Such potential may indicate a more extensive PV muscular sleeve, which required a greater number of radiofrequency applications for isolation and is more susceptible to late reconnection. In addition, (1) cPVB was seen in about 11% of PAF patients during SR prior to ablation, (2) cPVB was seen in about 3.5% of PV targeted at the index procedure and more frequently originate from the superior PV, and (3) cPVB is a useful marker for PV firing.

### 4.1. cPVB during Sinus Rhythm Prior to Ablation

Generally, a single PV potential closely following or fused with an atrial potential in the ostium of the PVs is the most commonly observed pattern during SR [7]. This study demonstrated that cPVB with a mean interval of 136 ± 16 ms was seen in about 11% of PAF patients during SR prior to ablation. The underlying mechanism of cPVB is yet to be defined. The origin of the first potential of the cPVB is most probably activation of a spiral PV myocardial sleeve from the LA. The origin of the second potential of the cPVB is less obvious. Reithmann et al. reported eight patients with pulmonary vein bigeminy, and 3 of them showed cPVB [13]. They considered the triggered activity or automatic activity to be the mechanism. Kim et al. reported two patients with PAF who demonstrate widely split PV double potentials at the PV ostium during SR [11]. In their study, the authors considered that the second potential is a result of slow conduction deep into the PV that returns and either reactivates the same myocardial sleeve at the ostium, also what is often considered spontaneous PV ectopy may actually be a result from reentry in and out of a PV with reactivation of the PV ostium and, when manifest, reactivation of the LA. In this study, the second potential may be a concealed PV ectopy which did not reactivate the LA. Even the appearance of the second potential after PV isolation was all coupled to the dissociated PVP. In these patients, the second potential is a result of slow conduction from the LA into a second PV fascicle is less likely. This dependent character strongly favors triggered activity as the arrhythmogenic mechanism. However, in some patients, we could not rule out a macroreentry in the vein or a slow conduction from the LA into a second PV fascicle may be the responsible mechanism because the second potential disappeared and never occurred again during the ostial ablation. The ostial ablation could partly destroy the circuit and prevent subsequent firing, eliminate the focus of PV ectopy, or interrupt the LA-PV conduction through the second PV fascicle.

### 4.2. cPVB and PV Firing

The current paradigm is that the PV sleeves primarily contribute to AF pathogenesis as a source of AF triggers [14]. In this study, 24 of 27 (89%) PVs with cPVB during SR showed focal firing spontaneously or with use of isoproterenol. When compared with PVs without cPVB during SR, the rate of focal firing was greater in PVs exhibiting cPVB (89% vs. 16%; *P* < 0.001). This observation may suggest that cPVB is a marker for PV firing during SR. In a postmortem anatomical study, Hassink et al. showed that patients with AF had significantly longer muscle sleeves present [15]. Similarly, muscle bundles in the superior veins were substantially longer than those in the inferior veins. Along with increased length, Guerra et al. using intravascular ultrasound found that patients with AF had considerably thicker PV myocardial tissue, and that PV firing could only be localized to these areas of thickening [16]. Nakagawa et al. also found that the incidence of PV firing increases with progressively wide LA-PV connections, and the number of radiofrequency applications required for isolation was greater in PVs exhibiting focal firing compared with PVs without firing [17]. In a study by De Greef et al., the incidence of preablation triggering PVs was higher in patients having recurrence of atrial fibrillation following the initial PV isolation compared with those who did not have a recurrence [18]. In this study, the distribution of the cPVB was more common in the upper veins compared with lower veins. The PV exhibiting cPVB also required a greater number of radiofrequency applications for isolation. All these characteristics are similar to that of dissociated pulmonary vein potentials after PV isolation reported by Lee et al. [19]. Compared to patients not exhibiting cPVB, the recurrence rate of AF in patients exhibiting cPVB was greater. All these similarities between cPVB and PV firing may indicate that cPVB itself is PV firing in a concealed manner which does not reactivate LA, that is to say “PV firing equals cPVB” to some extent. However, we could not rule out the role of the heart rate in the different results between patients with and without cPVB. When compared with the patients without cPVB, the higher heart rate in the patients with cPVB may represent the enhancement of the sympathetic nervous system which may cause a higher incidence of cPVB. The heart rate also can influence the contact between the catheter tip and myocardium which is important for lesion formation and may explain why a greater number of radiofrequency applications for isolation and a higher recurrence rate of AF in the patients with cPVB are needed. Jais et al. reported that the effective refractory periods of the PVs were shorter than that of the LA in AF patients but longer than LA in control patients, suggesting an association between short effective refractory periods in the PVs and the development of AF [20]. In this study, isoproterenol shortened the coupling interval of the cPVB, which is in accordance with clinical and experimental findings showing that isoproterenol shortens effective refractory periods and promotes the conduction in PV. Due to the relative shorter effective refractory periods in the PVs, the cPVB with possible mechanism of triggered activity or reentry mentioned above may be more susceptible to induce rapid PV firing. However, the activation sequence differed between cPVB and PV firing in all cases. The possible reason may be that cPVB and PV firing were conducted over the different PV fascicles. In addition, suppression of the second PV potentials by atrial pacing may explain the prevention of PAF by atrial pacing.

### 4.3. Clinical Implications

The present study was designed to obtain insight into the relation between cPVB and PV firing in PAF patients during SR and was not designed to define a new approach for ablation. The results of this study suggest that more extensive circumferential ablation may be required for the PAF patient exhibiting cPVB during SR.

## 5. Conclusion

cPVB during SR was observed prior to index ablation in 11% of PAF patients. Such potential itself may be a PV firing in a concealed manner which does not reactivate LA. The PV exhibiting cPVB required a greater number of radiofrequency applications for isolation. Compared to patients without cPVB, the recurrence rate of AF in patients with cPVB was greater.

## Figures and Tables

**Table 1:** Characteristics of cPVB.

	*N* (%)
Prevalence	
Total no. of patients (198)	22 (11%)
Total no. of veins (776)	27 (3.5%)
No. of cPVB/patient	1.2
PV potential location	
RSPV	9 (33%)
RIPV	3 (11%)
LSPV	10 (37%)
LIPV	5 (19%)
Wide double PV potentials interval	136 ± 16 ms
Relation to PV firing	24 (89%)

cPVB = concealed pulmonary vein bigeminy; PV = pulmonary vein; RSPV = right superior pulmonary vein; RIPV = right inferior pulmonary vein; LSPV = left superior pulmonary vein; LIPV = left inferior pulmonary vein.

**Table 2:** Patient demographics and procedural data.

Demographics	cPVB-positive (*N* = 22 patients)	cPVB-negative (*N* = 176 patients)	*P* value
Age (years)	65 ± 16	66 ± 20	0.462
Male (%)	64	62	0.917
Hypertension (%)	15	16	0.935
Diabetes (%)	5	4	0.856
AF history (years)	6.2 ± 5.1	6.9 ± 4.6	0.322
LA size (mm)	40 ± 6	41 ± 6	0.464
Weight (Kg)	76 ± 16	75 ± 12	0.605
Sleep apnea (%)	4	2	0.579
Structural heart disease (%)	6	6	0.943
Heart rate during sinus rhythm (beats/min)	72 ± 8	68 ± 8	0.002
RF number required for ipsilateral PVs isolation	21.6 ± 6.8	18.2 ± 5.6	0.024
AF recurrence (%)	27	17	0.032
Complication (%)	1	1	0.874
Follow-up (months)	33 ± 20	32 ± 20	0.713

cPVB = concealed pulmonary vein bigeminy; AF = atrial fibrillation; LA = left atrium; RF = radiofrequency.

## Data Availability

The data used to support the findings of this study are available from the corresponding author upon request.
